# Identification and Expression Profiling Analysis of the Cation/Ca^2+^ Exchanger (CCX) Gene Family: Overexpression of SlCCX1-LIKE Regulates the Leaf Senescence in Tomato Flowering Phase

**DOI:** 10.3389/fgene.2021.683904

**Published:** 2021-06-25

**Authors:** Jiao Li, Yaran Zhao, Chenliang Chang, Xin Liu, Jing Jiang

**Affiliations:** ^1^Key Laboratory of Protected Horticulture of Education Ministry, College of Horticulture, Shenyang Agricultural University, Shenyang, China; ^2^Vegetable Research Institute, Liaoning Academy of Agricultural Sciences, Shenyang, China

**Keywords:** CCX gene, bioinformatic analysis, expression patterm, leaf senescence, tomato

## Abstract

Cation gradients in plant cellular compartments are maintained by the synergistic actions of various ion exchangers, pumps, and channels. Cation/Ca^2+^ exchanger (CCX) is one of the clades of the Ca^2+^/cation antiporter super family. Here, five *SlCCX* genes were identified in tomato. Sequence analysis indicated that SlCCXs have the conserved motifs as the CCX domain. Analysis of the expression level of each member of tomato CCX gene family under cation (Mg^2+^, Mn^2+^, Na^+^, and Ca^2+^) treatment was determined by qRT-PCR. Tomato CCX demonstrated different degrees of responding to cation treatment. Changes in *SlCCX1-LIKE* expression was induced by Mg^2+^ and Mn^2+^ treatment. Analysis of the expression of *SlCCX* genes in different tissues demonstrated that constitutive high expression of a few genes, including *SlCCX1-LIKE* and *SlCCX5*, indicated their role in tomato organ growth and development. Overexpression of *SlCCX1-LIKE* dramatically induced leaf senescence. Transcriptome analysis showed that genes related to ROS and several IAA signaling pathways were significantly downregulated, whereas ETH and ABA signaling pathway-related genes were upregulated in overexpression of *SlCCX1-LIKE* (*OE-SlCCX1-LIKE*) plants, compared with the wild type (WT). Moreover, overexpression of *SlCCX1-LIKE* plants accumulated more ROS content but less Mg^2+^ content. Collectively, the findings of this study provide insights into the base mechanism through which CCXs regulate leaf senescence in tomato.

## Highlights

– Five *SlCCX* genes were identified in tomato.
– *SlCCX1-LIKE* was highly expressed in Mg^2+^ and Mn^2+^ treatment.
– Overexpression of *SlCCX1-LIKE* induced leaf senescence by increasing ROS, decreasing Mg^2+^, and hormone signaling pathways.

## Introduction

Calcium (Ca^2+^) is an essential macronutrient in plants owing to its unique role as a messenger (Hirschi, 2004). Spatiotemporal regulation of the cytosolic Ca^2+^ concentration is crucial for modulating cell responses to various internal and external stimuli (Dodd et al., 2010). Ca^2+^ influx and efflux, which are performed by transporters/channels, are regulated in a highly concerted manner, translating specific stimuli into a Ca^2+^ signal. In plants, Ca^2+^-permeable channels, Ca^2+^-ATPases, and Ca^2+^/cation antiporters (CaCA) mediate Ca^2+^ fluxes and maintain cytosolic Ca^2+^ homeostasis. Functional and phylogenetic analysis demonstrated that the CaCA superfamily comprises at least five gene families: the Na^+^/Ca^2+^ exchanger (NCX) family, the Na^+^/Ca^2+^, K^+^ exchanger (NCKX) family, the H^+^/cation exchanger (CAX) family, the YRBG family, and the cation/calcium exchanger (CCX) family (Liang et al., 1997; Cai and Lytton, 2004; Bonza and Michelis, 2011). Previous studies have suggested the evolutionary significance of CAs and CCXs in various plants due to the structural and functional similarity and divergence of these gene families (Emery et al., 2012; Singh et al., 2014). CCXs are a family of five Ca^2+^ flux proteins (*AtCCX1-5*) reported for *Arabidopsis thaliana* that were previously classified as members of CAX, namely *CAX7-CAX11* (Shimazaki et al., 2007). *AtCCX1* regulates leaf senescence and ROS homeostasis, and knockout of *AtCCX1* and *AtCCX4* showed stay-green leaf phenotypes that were sensitive to Ca^2+^ deprivation (Li et al., 2016). *AtCCX2* controls the dynamics of Ca^2+^ in the endoplasmic reticulum (ER), influencing plant growth in response to salt and osmotic stress (Corso et al., 2018). *AtCCX3* and *AtCCX4* have similar functions, because they arose from a single gene duplication (Morris et al., 2008). *AtCCX3* and *AtCCX4* are H^+^-dependent K^+^ transporters that may also ferry Na^+^ and Mn^2+^ (Liu et al., 1997; Tuttle et al., 2003), while *AtCCX5* might mediate K^+^ uptake and Na^+^ transport (Zhang et al., 2011). The *OsCCX2* gene, a rice member of this family, encodes a protein responsible for Ca^2+^, Na^+^, Li^+^, Fe^2+^, Zn^2+^, and Co^2+^ transport in certain yeast mutants (Yadav et al., 2015).

Leaf senescence is a highly integrated development stage that coordinates multidimensional alterations at the physiological and molecular levels. As a disintegrated and degenerated process, senescence is concomitant with an intensive restructuring of cells, involving the breakdown of macromolecules, such as chlorophyll, proteins, nucleic acids, and membrane lipid (Nam et al., 1980). During leaf senescence, intracellular organelles and micromolecules are dismantled and degenerated, which predominantly contributes to the source-to-sink allocation of nutrients (Noodé, 1988). The redistribution of nutrients from senescent organs to vegetative tissues is essential for plant fitness and critical for productivity and quality in crops (Lim et al., 2007). Generally, initiation of leaf senescence is age dependent and triggered by developmental cues (Woo et al., 2019).

Intrinsically, initiation of senescence is the consequence of integrated signals, including endogenous and environmental signals (Bresson et al., 2018). Developmental senescence, which is a coordinated physiological process, could be induced by the endogenous factors (Zentgraf et al., 2004). Ca^2+^ has a regulatory role in vegetative senescence and fruit ripening (Ferguson, 1984). The impairment of leaf cell Ca^2+^ uptake in leaf cells hastens senescence programming (Ma et al., 2010). Furthermore, phytohormones play both positive and negative roles in the onset of leaf senescence. Among the leaf senescence-related hormones, abscisic acid (ABA), auxin, and ethylene (ETH) can significantly accelerate the aging of leaves (Li et al., 2013). In addition to hormone responsive genes, numerous senescence-related genes, such as senescence-associated genes, have been proven to be crucial for the timing of leaf senescence (Park et al., 1998).

Calcium is an essential component involved in plant senescence signaling cascades. As the cation conducting channel, CNGC2 is involved in leaf senescence signaling. Regarding the CCX gene family, we found that the expression of SlCCX1-LIKE varies dramatically across different organs in tomato. In addition, further study showed that leaf senescence was increased in SlCCX1-LIKE-OE plants. In our experiment, SlCCX1-LIKE was selected as the candidate gene for the study through the analysis and identification of the biological information of the members of SlCCXs and the phenomenon of leaf senescence induced by SlCCX1-LIKE-OE. Hence, our findings provide further theoretical basis for the mechanism of leaf senescence, which may have important agricultural implications on yield and nutrition content as affected by CCXs.

## Materials and Methods

### Identification of Members of the SlCCX Family in Tomato

To identify tomato *SlCCXs*, *Arabidopsis CCX* protein sequences were used as queries. A BLASTP search was performed to identify members of the *SlCCXs* in tomato against the Solanaceae Genomics Network database^1^. These genes were termed *Solanum lycopersicum CCXs* (*SlCCXs* which contain *SlCCX1*; *SlCCX1-LIKE*; *SlCCX4*; *SlCCX4-LIKE*; *SlCCX5*), and the sequences were further used as queries for the BLASTN searches, against SGN tomato whole genome scaffolds data (2.10) (International Tomato Genome Sequencing Consortium)^2^. The *SlCCXs* were further used as query sequences to search for CCXs of the other Solanaceae members (Supplementary Table 1), using the NCBI^3^ and SGN^4^.

### Basic Features, Secondary Structures, and Subcellular Localization

The molecular mass, theoretical isoelectric point, instability coefficient, and hydrophilicity index of *SlCCXs* were compiled by analyzing the physical and chemical properties of the amino acids using the ExPASy ProtParam tool^5^. The deduced amino acid sequences of the *SlCCXs* were submitted to TMHMM Server v.2.0^6^ in FASTA format, using the default settings to predict the presence of protein domains. Structural predictions were performed using the structural functional domain of the Simple Modular Architecture Research Tool (SMART)^7^. Subcellular localization was performed using the PSORTII Prediction program^8^.

### Sequence Alignment, Gene Structure, and Phylogenetic Analyses

Multiple sequence alignments for the *SlCCXs* were generated using ClustalW in DNAMAN 8.0 Demo sequence analysis tool, and the phylogenetic tree was constructed with the neighbor-joining algorithm in MEGA7.0 (Kumar et al., 2016). The neighbor-joining (NJ) method was applied to construct a phylogenetic tree, in which Poisson correction, pairwise deletion, and bootstrapping (1,000 replicates; random seeds) were used as the default values to evaluate the liability of the tree.

A schematic diagram of the gene structure of *SlCCXs* was constructed using the Gene Structure Display Server^9^. To identify the conserved motifs of the *SlCCX* protein sequences, we used the online multiple expectation maximizations for motif elicitation (MEME) tool^10^.

Sequence domains were identified through the Pfam 32.0 database collection^11^. Genes that did not contain the known conserved domains and motifs of the *SlCCX* members were eliminated using the SMART database^12^ and the Conserved Domain NCBI database^13^ (Yue et al., 2012). Genomic information concerning the chromosome locations of the *SlCCXs* and the amino acid sequences of *SlCCX* proteins was obtained from the SGN database.

### qRT-PCR Analysis, Tomato Plant Growth, and Treatments

Total RNA from the samples of leaves was extracted using TRIzol^®^ (Takara, Dalian, China). Genomic DNA contamination was removed followed by RQ1 DNase I (Promega, Madison, WI, United States). cDNA was synthesized with DNA-free RNA (2 μ). The RT-PCR for target mRNA was performed according to Liu et al. (2016). Primers are listed in Supplementary Table 2.

Tomato (*S. lycopersicum* “Micro-Tom”) seedlings were planted in pots containing the growth medium (nutrition soil:vermiculite:perlite = 3:2:1) and grown under a 12-h/12-h (light/dark) photoperiod at 25°C, a relative humidity of 70–80%, and an irradiance of 300 μmol m^–2^ s^–1^. Fruits were harvested at both maturing stage (green) and ripening stage (red). Fruit pericarp disks were prepared with a cork borer (4 mm in diameter and 2 mm in thickness) and equilibrated for 30 min in equilibrating buffer (MES 50 mM at pH 5.5, containing 5 mM CaCl_2_, 5 mM MgCl_2_, 5 mM EDTA, 5 mM vitamin C, and 200 mM mannitol) (Télef et al., 2006; Marchler-Bauer, 2011). Mannitol was used to adjust the equal osmotic potential in the incubation system. After equilibration, disks in the control untreatment were incubated in equilibration buffer, while the other disks were incubated in equilibration buffer with 50, 100, and 200 mM CaCl_2_, MgCl_2_ (Aliu et al., 2015), NaCl, or MnCl_2_ (Xu et al., 2010) for 8 or 24 h (Jia et al., 2016) at room temperature (25°C–28°C). After being washed by double distilled water, the tissues were used immediately for assays or frozen in liquid nitrogen and kept at −80°C until use. Experiment was repeated at least three times. Each experiment consisted of three biological replicates and three technical replicates.

For H_2_O_2_ treatment, the fourth and fifth detached leaves from about 35-day-old plants were incubated on 30 mM H_2_O_2_ solutions for 4 days in the dark.

### Digital Expression Analysis of CCX Genes in Growth and Development

The expression analysis of *SlCCX* genes were downloaded from the Tomato Functional Genomics Database^14^, including sequence data from various tissues in tomato cultivar “Heinz 1706” (*S. lycopersicum*) and wild species LA1598 (*Solanum pimpinellifolium*). *SlCCX* genes expressed in leaves, roots, unopened flower buds, fully opened flowers, and at different stages of fruit development (1, 2, and 3 cm fruit, mature green fruit, breaker fruit, and fruit at 10 days after breaker) were summarized and used to construct a heatmap with Multiple Array Viewer software to visualize the expression profiling based on log2-transformed RPKM values.

### Generation of Transgenic Tomato Plants Overexpressed SlCCX1-LIKE

The full-length coding sequence of SlCCX-LIKE was amplified by PCR and then inserted into a pCAMBIA3301 vector to construct overexpression vector. Harboring *SlCCX-LIKE* vector was transformed into *Agrobacterium tumefaciens* GV3301. Putative hardened transgenic plants were transferred to the greenhouse. Presence of the transgene was confirmed by PCR with kanamycin (Kana) primers. Seeds from the transformants were grown on half-strength Murashige-Skoog (MS) medium supplemented with 100 mg L*^–^*^1^ kanamycin for selection. Surviving seedlings were grown in a greenhouse to obtain the T1 generation. Four OE lines were selected for subsequent analysis.

### Determination of Chlorophyll Content and ROS Concentrations

Tissues were homogenized in liquid nitrogen. The powdered samples were extracted with 80% acetone. Chlorophyll content was determined spectrophotometrically at wavelengths of 663 and 645 nm.

The H_2_O_2_ and O_2_^⋅–^ was measured using hydrogen peroxide (H_2_O_2_) Content Assay Kit (Solarbio, Beijing, China) and Superoxide Anion Activity Assay Kit (Solarbio, Beijing, China) as per manufacturer’s instructions.

### Ca and Mg Determination

Leaves were dried in an oven at 100°C for 1 h, and then 65°C for 2 days. The dried samples were weighed and digested with H_2_SO_4_ solution at 180°C for 10 min, then the temperature was adjusted to 220°C for 30 min. The concentrations of Ca^2+^ and Mg^2+^ were determined using Atomic Absorption Spectrophotometer 6300 (Agilent, Santa Clara, CA, United States). Experiment was repeated at least three times. Each experiment consisted of three biological replicates and three technical replicates.

### RNA-seq Library Construction and Transcriptome Analysis

RNA extracts (three biological replicates for each of two genotypes), each containing at least 5 mg of total RNA, were sent to a private genomic laboratory, GENEWIZ, for RNA sequencing. Raw reads containing adapters, >10% of unknown nucleotides, and low-quality reads (*Q*-value ≤ 20; >50% of reads) were removed from the dataset. To estimate gene expression level, trimmed reads were mapped to the tomato genome CDS (ITAG release 4.0). Reads mapped to unigenes were counted and used for expression analysis.

### Subcellular Localization Assays in Tobacco Leaf Cells

The 1.5-kb promoter sequence upstream of ATG and the SlCCX1-LIKE coding region were inserted into the pCAMBIA2300-GFP vector by SacI and XbaI digestion and using the 35S promoter. Recombinant plasmids were transformed into *A. tumefaciens* GV3101 cells. After grown in LB medium at 28°C overnight, bacterial suspensions were infiltrated into young but fully expanded leaves of tobacco using a needleless syringe. After infiltration, plants were immediately covered with plastic bags and placed at 23°C for 48 h before bag removal. The GFP fluorescence signals were observed with a confocal laser scanning microscope (Leica SP8, Germany). The fluorescence signal was observed at an excitation wavelength of 488 or 561 nm and emission wavelength of 500–572 or 605–635 nm.

## Results

### Identification of SlCCX Family Members

In this study, we identified five *SlCCX* genes in tomato named according to their homologous recombination in *A. thaliana.* Gene name, gene accession number, locus, protein sequence length, molecular weight, and isoelectric point (p*I*) are listed in Table 1.

**TABLE 1:** CCXs gene information in tomato.

Gene name	Gene accession number	Locus	Protein length (AAs)	Molecular weight (kDa)	Isoelectric point	Instable coefficient^*a*^	The grand average of hydropathy^*b*^
*SlCCX1*	Solyc09g072690.1.1	LOC101248713	568	62.67	6.31	37.00	0.703
*SlCCX1-LIKE*	Solyc07g006370	LOC101250521	567	62.42	8.00	33.31	0.706
*SlCCX4*	Solyc02g069710.2.1	LOC101265300	476	52.42	5.91	42.66	0.370
*SlCCX4-LIKE*	Solyc07g042000	LOC101250709	646	70.02	5.73	34.73	0.608
*SlCCX5*	Solyc01g098800	LOC101261070	555	60.84	6.49	32.67	0.868

*^*a*^Instable coefficient: a protein with an instability coefficient of less than 40 is called a stable protein.*

*^*b*^The grand average of hydropathicity is calculated by adding the hydropathy value for each residue and dividing by the length of the sequence.*

We found that length of the nucleotide chain of members of the *SlCCX* gene family was between 476 and 646 bp, with the shortest and longest being *SlCCX4* and *SlCCX4-LIKE*, respectively. The molecular weight of SlCCX4 was 52.42 kDa, and for the SlCCX4-LIKE, it was 70.42 kDa. After analyzing the physicochemical properties of these proteins, we found that the theoretical isoelectric pI of the amino acids is between 5.73 and 8.00, which we refer as intermediate, with only the SlCCX1-LIKE having a p*I* of 8.0. Among the identified members, the GRAVY value of SlCCX5 was the highest (Table 1).

**FIGURE 1:**
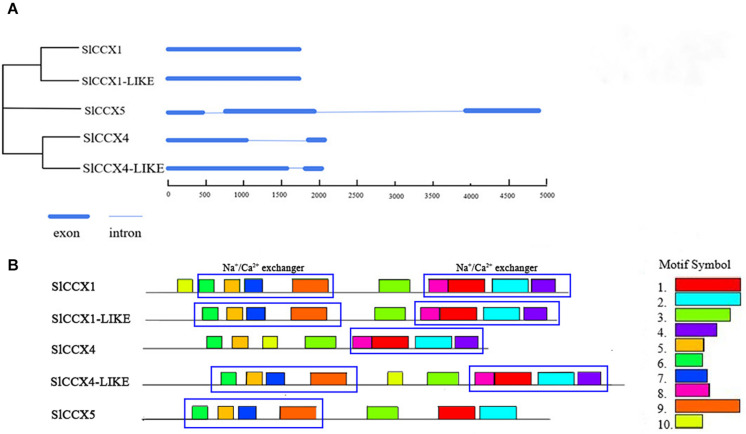
Exon, intron, and motif analysis of *SlCCXs.* **(A)** The neighbor-joining phylogenetic analysis of the CCX subfamily in tomato. **(B)** Distribution of conserved motifs of CCX proteins in tomato. Different motifs are indicated by different colors numbered 1–10. The same number in different proteins refers to the same motif. The conserved Na^+^-Ca^2+^ exchanger are shown in blue-colored boxes.

*SlCCX1* and *SlCCX1-LIKE* are composed with no introns, while *SlCCX4* and *SlCCX4-LIKE* have one intron. In contrast, *SlCCX5* has three exons and two introns. Motifs present in the five SlCCX proteins were identified using their full-length protein sequence from tomato (Figure 1). A total of 10 conserved motifs were identified, and their distribution in proteins of the respective groups are presented in the phylogram in Figure 1. Motif 7 corresponds to α1 signature domain (GNGAPD), and motif 1 corresponds to α2 signature domain (G(N/D)SxGD). SlCCX1, SlCCX4-LIKE, and SlCCX1-LIKE and SlCCX5 have 10, nine, and eight motifs, respectively. Motifs 1, 2, 3, 5, and 6 were the most conserved, as shown by their presence in all SlCCX protein members. However, motifs 3, 5, 7, and 9 constitute the Na^+^/Ca^2+^ exchanger domain; while motifs 8, 1, 2, and 4 constitute the Na^+^/Ca^2+^ exchanger. Moreover, motifs 4 and 8 were not found in SlCCX5, and motif 7 was not found in SlCCX4. This suggests that the SlCCX4 and SlCCX5 have one Na^+^/Ca^2+^ exchanger domain, whereas SlCCX1, SlCCX1-LIKE, and SlCCX4-LIKE have two Na^+^/Ca^2+^ exchanger domains. In contrast, SlCCX4 lacks motif 7 containing a conserved sequence within the α1 repeat regions. SlCCX4 and SlCCX4-LIKE motifs were similar and both lacked motif 10. This feature may affect the spatial configuration of the protein, which might affect its cation transport-related characteristics.

### Phylogenetic Analysis of SlCCXs

Using query CCX protein query sequences from tomato, we performed a genomewide analysis to identify SlCCX (CCX in *S. lycopersicum*) members from eight *Solanum* genomes (*Solanum pennellii*, *Solanum tuberosum*, *Nicotiana attenuata*, *Nicotiana tabacum*, *Capsicum annuum*, *Nicotiana sylvestris*, *Capsicum baccatum*, *Capsicum chinense*). The characteristics of the databases we used in this research, as well as the number of genes, are shown in Supplementary Table 1. The nomenclature used for all CCXs from the different members of the Solanaceae family is similar to that used for SlCCXs, e.g., *S. tuberosum CCX* (*StCCX*) genes, *Nicotiana tomentosiformis CCX* (*NtCCX*) genes, and *N. sylvestris CCX* (*NsCCX*) genes.

**FIGURE 2:**
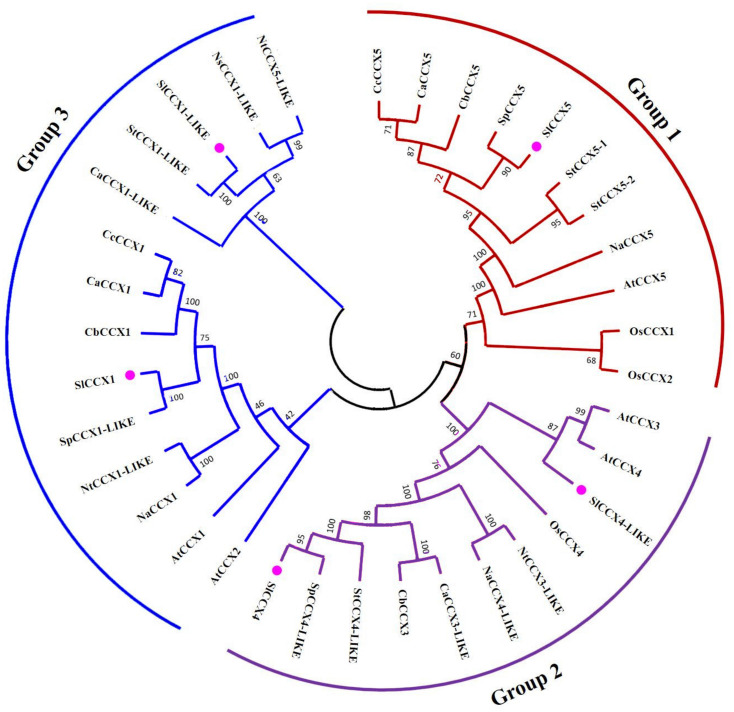
Phylogenetic analysis in Solanaceae CCXs. Multiple sequence alignment of CCX proteins was done using DNAMAN, and phylogenetic tree was constructed using MEGA7.0 by the neighbor-joining method with the pairwise deletion option with Poisson correction. The number at each node represents the bootstrap value from 1,000 replicates. *At*, *Arabidopsis thaliana*; *Sp*, *Solanaceae pennellii*; *St*, *Solanaceae tuberosum*; *Na*, *Nicotiana attenuata*; *Nt*, *Nicotiana tabacum*; *Ca*, *Capsicum annuum*; *Cb*, *Capsicum baccatum*; *Cc*, *Capsicum chinense*; symbol indicates tomato SlCCX proteins.

We constructed the unrooted phylogenetic tree containing 28 CCX protein sequences from various members of the Solanaceae family (five SlCCXs, two CcCCXs, four CaCCXs, three CbCCXs, three SpCCXs, four StCCXs, three NaCCXs, three NtCCXs, and one NsCCX) was constructed by aligning them with three sequences from *Oryza sativa* (OsCCX) and five sequences from *Arabidopsis* AtCCXs, using MEGA7.0 tools. These CCX sequences were categorized into three groups (Figure 2), groups 1, 2, and 3. A total of 13 sister pairs, including three S1CCX-SpCCX, two NtCCX-NaCCX, two CaCCX-CcCCX, and one each of StCCX-StCCX, OsCCX-OsCCX, AtCCX-AtCCX, CaCCX-CbCCX, StCCX-SlCCX, and NtCCX-NsCCX pairs are present in the combined phylogenetic tree. The data indicate a high homology within the Solanaceae family. The two proteins with (1–10-motif) domains (SlCCX4 and SlCCX4-LIKE) were clustered together in group 3. SlCCX1 and SlCCX1-LIKE without the 10-motif domain were listed in group 2. Group 1 consists of the SlCCX5 without the four-, eight-, and 10-motif domains.

### Analysis of the SlCCX Protein Structure

**FIGURE 3:**
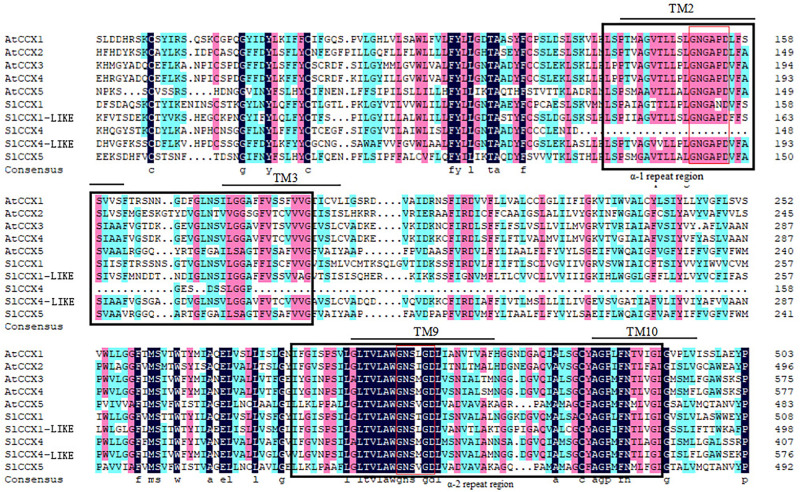
Multiple sequence alignment of CCX proteins. Figure shows alignment of CCX protein sequences of *Arabidopsis* and tomato. Conserved α1- and α2-repeat regions are shown in black-colored boxes. The common conserved domain “GNG(A/S)PD” in α1-repeat and “G(N/D)SxGD” in α2-repeat motif are shown in red lines. The predicted transmembrane (TM) spans are shown in black color lines.

SlCCX putative protein sequences and their secondary structures are shown in Table 2 and Figure 3. In general, SlCCXs were characterized by α-helices, extended chains, random coils and β-turns, where the angle of β-turn accounted for the lowest proportion. The random curl in SlCCX4 was larger than the α-helix, while the other four proteins had irregular curls that were smaller than the α-helix.

**TABLE 2:** Secondary structures analysis of the *SlCCXs*.

Protein	Proportion%			
	α -Helix	Extended chain	Random coil	β -Turn
SlCCX1	30.99	31.69	27.64	9.68
SlCCX1-LIKE	29.28	33.16	27.69	9.88
SlCCX4	30.88	26.26	32.98	9.87
SlCCX4-LIKE	30.65	30.19	29.10	10.06
SlCCX5	39.82	22.88	27.75	9.55

*Proportion is the percentage of all the parts.*

Most CCXs had 10–12 transmembrane (TM) helices, with two conserved alpha repeats in the TM helices 2–3 and 7–8. The *S. lycopersicum* ion-exchanger sequence likely contains the α-1 and α-2 repeat regions, as evidenced by the presence of domains having 100% identity with the α-1 signature motif GNG (A/T) PD and the α-2 signature motif GNSIGD (Figure 3).

**FIGURE 4:**
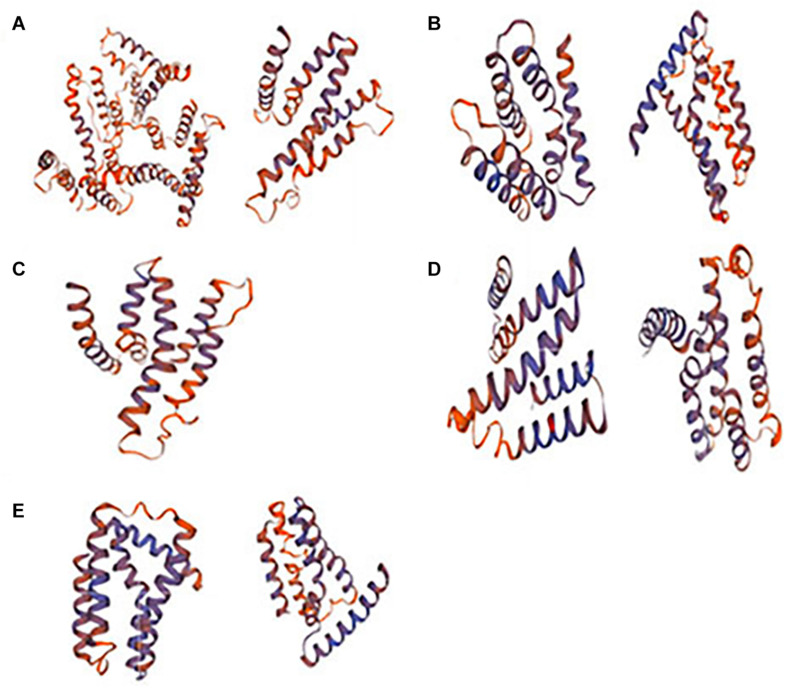
Modeling structure of the Na^+^/Ca^2+^ exchanger domains in SlCCXs. Note: **(A–E)** SlCCX1, SlCCX1-LIKE, SlCCX4, SlCCX4-LIKE, and SlCCX5.

Protein modeling analysis verify the Na^+^/Ca^2+^ exchanger domain in SlCCXs (Figure 4). Gene structure analysis showed that all four genes have two Na^+^/Ca^2+^ exchanger and the Na^+^/Ca^2+^ transporter regions, while SlCCX4 has only a conserved Na^+^/Ca^2+^ exchange region.

### Expression Pattern of *SlCCX* Genes in the Tomato and Under Various Cation Treatment Conditions

**FIGURE 5:**
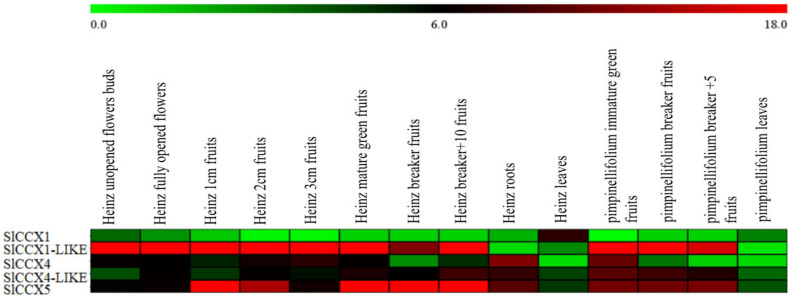
Heatmap showing differentially expressed SlCCX1-LIKE. Note: Red represents high expression, green represents low expression, and the expression in the figure represents the actual expression of log2 value. From left to right is Heinz unopened flowers buds; Heinz 1 cm fruits; Heinz 2 cm fruits; Heinz 3 cm fruits; Heinz mature green fruit; Heniz breaker fruits; Heniz breaker + 10 fruits; Heinz roots; Heniz leaves; *S. pimpinellifolium* immature green fruits; *S. pimpinellifolium* breaker + 5 fruits; *S. pimpinellifolium* leaves.

Compared with their paralogs, three genes (*SlCCX1-LIKE*, *SlCCX4-LIKE*, and *SlCCX5*) showed significantly higher levels of expression (compared with their two remaining paralogs) in the different organs of the tomato cultivar “Heinz1706” and the wild relative *S. pimpinellifolium*. *SlCCX1-LIKE* gene (from group 3) was highly expressed in reproductive organs, such as flowers and developing fruits, but showed reduced expression levels in the vegetative organs, such as roots and leaves. *SlCCX5* expression in group 1 remained constant and high during fruit ripening. *SlCCX4-LIKE* from group 2 was found to be preferentially expressed in roots and ripening fruits (Figure 5).

**FIGURE 6:**
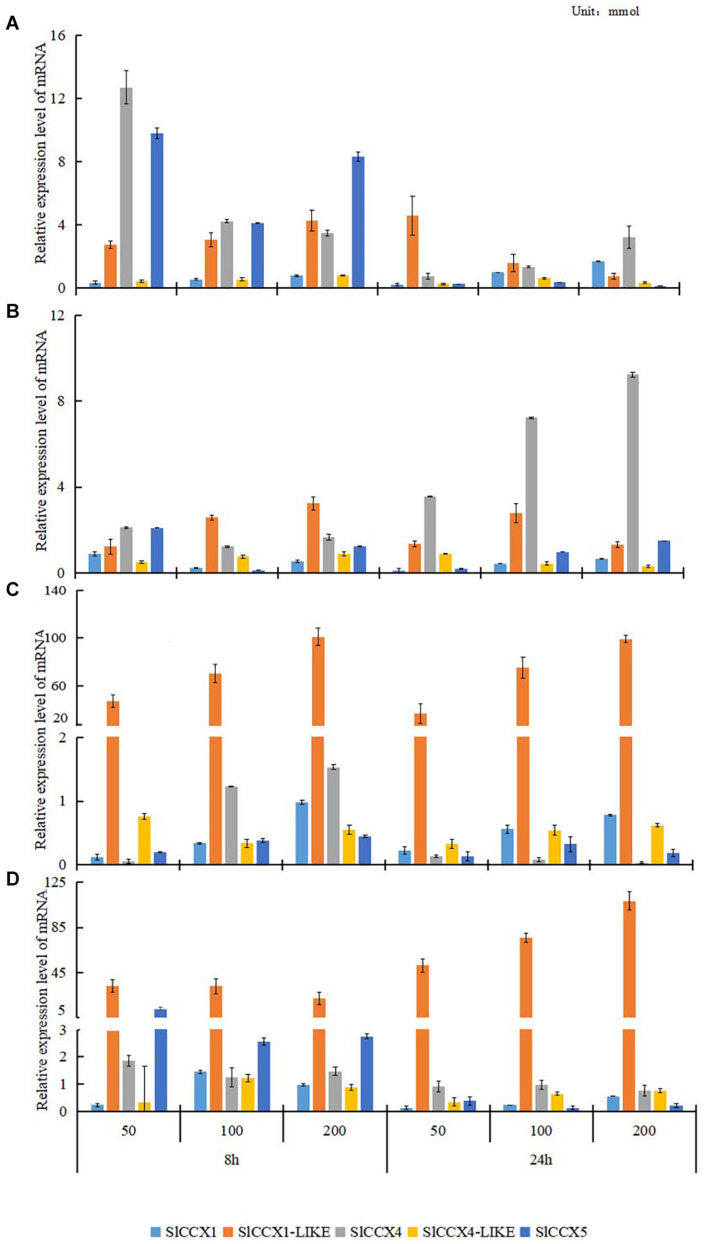
The expression of SlCCXs under different cation concentrations treatments in mature-green fruits. **(A–D)** Different ion treatments: **(A)** NaCl; **(B)** CaCl_2_; **(C)** MgCl_2_; and **(D)** MnCl_2_. The treatment concentration of each ion was 50, 100, and 200 mM. The treatment time was 8 and 24 h. Bar represents the corrected standard and error. Data were normalized to *ACTIN* gene expression, and values of relative expression data of untreatment control (0 h, 0 mM) was normalized to 1.

To evaluate the role of *SlCCX* genes under various cation treatments, we determined their expression levels in the fruits of the “Micro-Tom” tomato under cation treatments. The expression of *SlCCX* genes was significantly affected by different cation treatments (Figure 6). At the mature green stage, the expression levels of *SlCCX1-LIKE* were much higher in fruits exposed to MgCl_2_ and MnCl_2_ than those in the control treatment, and the extent of *SlCCX1-LIKE* upregulation increased significantly with the increasing cation concentration and exposure time (Figure 6). Expression levels of the other *SlCCX* family members were similarly low in tissues exposed to different cation treatments. Compared with the control, the expression level of *SlCCX1* and *SlCCX4-LIKE* genes were low under the different cations and exposure time treatments. *SlCCX4* exhibited maximum expression after tissues were exposed to 50 mmol for 8 h. The expression of *SlCCX4* increased significantly after 24 h of exposure to CaCl_2_, compared with that after 8 h. Contrastingly, the expression level of *SlCCX5* was higher at 8 h than at 24 h under NaCl and MnCl_2_ treatments. *SlCCX1-LIKE* showed higher expression under the divalent cation treatments in ripening organs. Ion transport, mostly of divalent cations, may play an important role in organ developing ripening.

### Overexpression of SlCCX1-LIKE Promotes Leaf Senescence in Tomato

**FIGURE 7:**
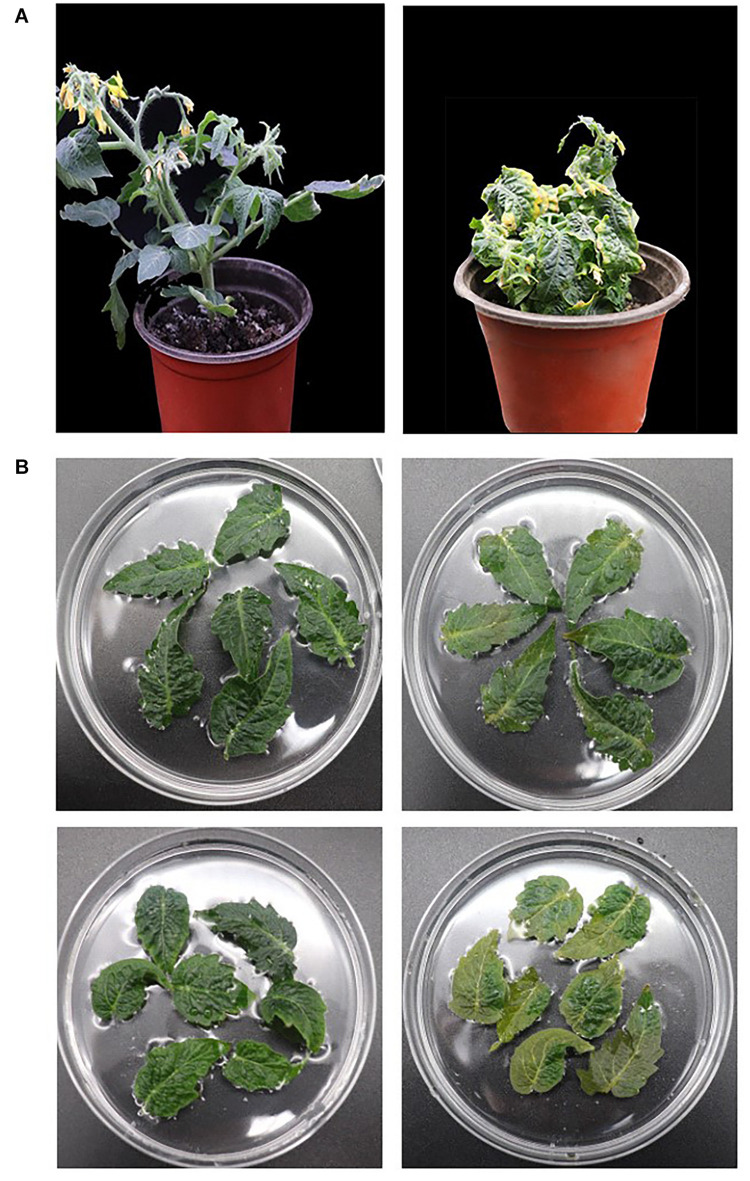
Phenotype of the leaf senescence in SlCCX1-LIKE-OE plants. **(A)** The whole WT (left) and SlCCX1-LIKE-OE (right) transgenic plant. **(B)** Phenotypes of leaves with H_2_O_2_ treatment. The detached leaves of WT (left) and SlCCX1-LIKE-OE (right) plants were treated with H_2_O_2_ at 1 and 4 days.

After bioinformatics and expression analysis above, SlCCX1-LIKE was selected for further investigation. SlCCX1-LIKE was overexpressed in “Micro-Tom” tomato. SlCCX1-LIKE-OE-3, OE-4, and OE-6 plants were selected from the T1 generation plant for the transgenic stable genetic technology (Supplementary Figure 1). Thirty-day-old seedling leaves turned yellow earlier than WT (Figure 7A). To explore further the involvement of SlCCX1-LIKE-OE in ROS homeostasis, we examined the ROS tolerance of WT and OE plants to ROS by treating detached leaves from 35-day-old seedings with H_2_O_2_ (Figure 7B). SlCCX1-LIKE-OE leaves turned yellow at the 4 days treatment. In contrast, the WT leaves were still green after 4 days treatment of H_2_O_2_. Therefore, compared with WT, SlCCX1-LIKE-OE tomato seems more sensitive to H_2_O_2_ treatment.

### Expression of Hormone-Responsive Genes and ROS-Related Gene Expression in OE Plants

**FIGURE 8:**
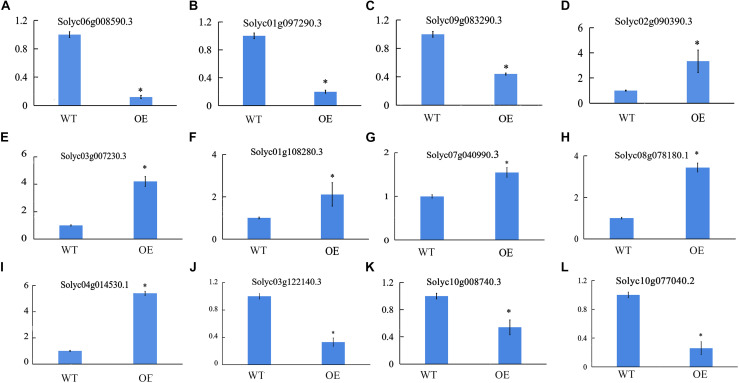
Quantitative real-time PCR validation of differentially expressed genes. **(A–C)**, IAA response/signal-related genes; **(D–F)** ETH response/signal-related genes; **(G–I)** ABA response/signal-related genes; **(J)** ROS signal-related genes; **(K)** and **(L)**, Mg^2+^ related genes. *Significant differences with *P* < 0.05 determined using a Duncan’s test compared with the control.

We also sequenced the RNA from the fifth leaves of WT and OE-SlCCX1-LIKE plants. To validate the RNA sequencing results, 12 genes that were annotated in GenBank were selected for qRT-PCR analysis (Figure 8). The results suggest that the RNA sequencing and qRT-PCR results are similar expression tendencies, confirming that the RNA sequencing results were reliable and could be used for further analysis.

Approximately 32% of the differentially expressed genes were found to be involved in hormone processes. In total, 168 differentially expressed gene (DEGs) were classified to be involved in the hormone category, which was subclassed into the top three groups (IAA53%, ABA16%, ETH14%) (Supplementary Table 3). We also found 24 DEGs related to auxin-induced SAUR-like protein, 11 IAA family genes, and six GH3 (Gretchen Hagen 3) family protein. All these DEGs were all downregulated in OE plants. In contrast. five ethylene-responsive transcription factors, two ethylene-insensitive proteins, and three ethylene receptors were all upregulated in OE plants. Two protein phosphatase 2C and one serine/threonine-protein kinase related to ABA signaling transduction were also upregulated in OE plants (Supplementary Table 3). Eleven genes related to the reactive oxygen scavenging enzyme system were also downregulated in OE plants. This indicates that ROS content in SlCCX-LIKE-OE leaves might be increased. Mg^2+^-related enzyme genes were also downregulated in SlCCX-LIKE-OE leaves. The Mg^2+^ signal may be influenced by SlCCX1-LIKE.

### Leaf Senescence in OE Plants

**FIGURE 9:**
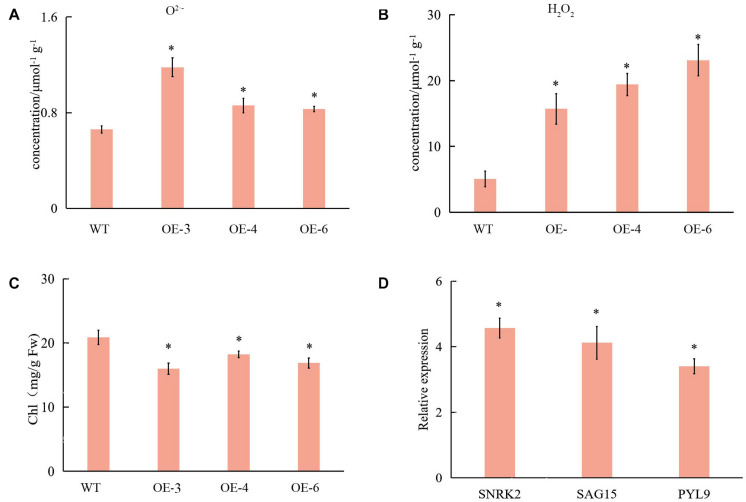
The content of the O_2_^⋅–^ **(A)** and H_2_O_2_ **(B)** in WT and OE plant of 25 days seedlings. Chl content **(C)** and relative expression of WT and OE plants related with leaf senescence **(D)**. SNRK 2, sucrose non-fermenting 1-related protein kinase 2; SAG15, senescence-associated gene15; PYL, pyrabactin resistance 9. Relative expression levels of leaves senescence with OE plants compare with control (WT). The experiments were repeated three times. Error bars indicate the means ± SE of three independent replicates. Expression data of WT was normalized to 1. *Significant differences with *P* < 0.05 determined using a Duncan’s test compared with the control.

Transcriptome data showed that ROS-related genes were affected in OE plants and WT; therefore, we determined their ROS content. The H_2_O_2_ and O_2_^⋅–^ contents were significantly higher in SlCCX1-LIKE, compared with WT (Figures 9A,B). These results suggest that the ROS was induced in SlCCX1-LIKE-OE plants.

**FIGURE 10:**
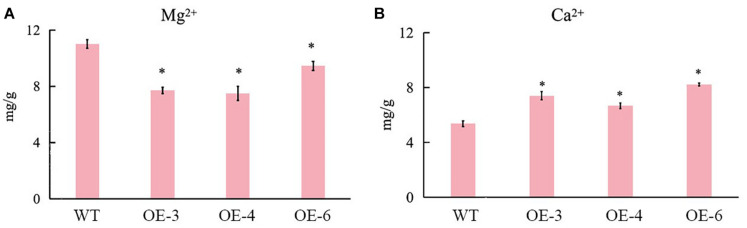
The concentration of **(B)** Ca^2+^ and **(A)** Mg^2+^ in WT and SlCCX1-LIKE-OE plants of 25 days seedlings. The experiments were repeated three times. Error bars indicate the means ± SE of three independent replicates. *Significant differences with *P* < 0.05 determined using a Duncan’s test compared with the control.

There were no distinguishable growth and development phenotypic differences between SlCCX1-LIKE-OE and the WT. However, we observed that chlorophyll content was significantly downregulated during natural senescence in SlCCCX1-LIKE-OE plants (Figure 9C). The senescence-related genes were also induced in SlCCX1-LIKE-OE plants (Figure 9D). Magnesium is a major component in chlorophyll and an activator of many enzymes. If calcium content declines, magnesium may replace some of the calcium. Therefore, downregulation of magnesium ions affects leaf growth and accelerates leaf aging. Mg^2+^ concentration was decreased in the OE plants (Figure 10). These results indicated that SlCCX1-LIKE-OE regulated the Ca^2+^ and Mg^2+^ concentration to accelerate the aging of leaves in the OE plants.

### Subcellular Localization of SlCCX1-LIKE

**FIGURE 11:**
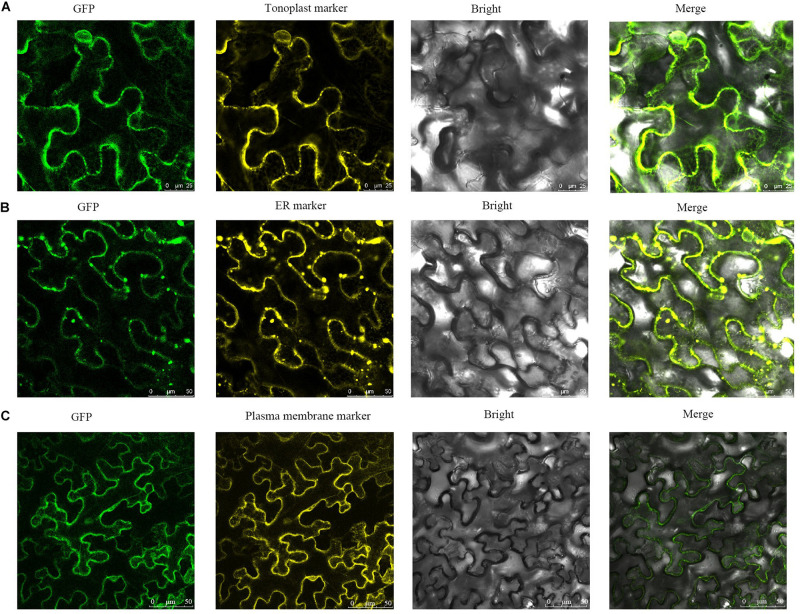
Subcellular localization of SlCCX1-LIKE in the leaves of *Nicotiana benthamiana*. **(A)** Tonoplast maker; **(B)** ER marker; **(C)** plasma membrane marker.

AtCCX2, a member of the *Arabidopsis* CCX subfamily, regulates Ca^2+^ concentrations in the ER, while AtCCX3 and AtCCX5 are localized to the endomembrane system (Morris et al., 2008). To test CCX family localization in tomato, the *SlCCX1-LIKE* was chosen as representative member and fused to GFP for the subcellular localization analysis (Figure 11). The transient gene expression assay showed that SlCCX1-LIKE, a putative cation/Ca^2+^ exchanger, may be localized to the tonoplast, ER, and plasma membrane.

## Discussion

Recently, extensive research has focused on the regulation of cytosolic calcium concentration and its response to the internal and external stimuli, during growth and developmental processes in plants (Cai and Lytton, 2004; Hirschi, 2004; Taneja et al., 2016). A recent study analyzing the *A. thaliana* genome identified 10 CCX genes sharing high similarity with known CCXs from other species (Mäser et al., 2001). In the present study, five of the CCX genes were characterized in tomato. Their sequence and expression analyses will contribute to a better understanding of this gene family and provide potential functional characterization of the important members of the CCX family in tomato.

### Functional Differences Related to SlCCX Family Structure Characteristics

In this study, we identified five CCX genes in tomato. From the analysis sequence length, p*I*, and MW (Table 1), we verified that the CCX genes of tomato share the similarities with *Arabidopsis* and rice, suggesting functional similarity and an evolutionarily conserved nature. While two, four, three, three, four, one, three, and two orthologous proteins from other species of the Solanaceae family (namely *S. pennellii*, *S. tuberosum*, *N. attenuata*, *N. tabacum*, *C. annuum*, *N. sylvestris*, *C. baccatum*, and *C. chinense*, respectively) were constructed with the NJ method, dividing all CCXs into three well-supported groups (Figure 2), consistent with a previous study (Blatt, 2000). It has been demonstrated that structural divergence plays important roles in the evolution of multiple gene families, mainly through exon/intron gains/losses, exonization/pseudoexonization, and insertion/deletion (White and Broadley, 2003). These genes are relatively conserved within the Solanaceae family (including tomato) without the likelihood of large-scale duplication events in the past. According to the phylogenetic tree, the *SlCCX1* and *SlCCX1-LIKE* genes from group 3 contained only one, *SlCCX4* and *SlCCX4-LIKE* genes contained two, and the SlCCX5 contained three exons. The exons of the homologous genes tends to remain conserved during evolution. The five SlCCXs members are in a separated branch of the phylogenetic tree without any CCX members from *Arabidopsis*, except for the SlCCX4-LIKE grouped with AtCCX4. This may indicate that the other four *SlCCX* genes were specific to Solanaceae plants. Due to a high degree of conservation within the Solanaceae family, the identification and characterization of *SlCCX* genes from tomato will contribute in identifying these genes and characterizing their putative group function in other Solanaceae species.

In this study, we identified and characterized conserved motifs in the SlCCX family in tomato by the MEME. Most SlCCX proteins clustered in the same group sharing similar motifs, suggesting that these conserved motifs play crucial roles in the group functions. Multiple alignments of SlCCX sequences revealed the presence of several, highly conserved sequence motifs. One of a particular importance is the [G (N/D)SxGD] motif (motif 1 in this study), which is referred as “α2 signature domain” and is critical for the function of the CCX transporters. The specificity of the CCX family member motifs leads to different function in maintaining ion homeostasis. AtCCX1 and AtCCX2, for example, are involved in the transport of Ca^2+^, while AtCCX3 and AtCCX5 are involved in the transport of Na^+^ and K^+^ but not Ca^2+^ (Sze et al., 2000; Hirschi, 2004; Li et al., 2016). Ten motifs are located differently in five SlCCXs. This might affect the spatial configuration and consequently the characteristics of the cation transport of the five members. Motif distribution analysis confirmed a conserved nature but a high functional divergence among CCX proteins. This finding corroborated the outcome of the phylogenetic analysis.

### SlCCXs Expression Pattern Mediating the Ion Response and Hormone Signals

Differential SlCCX expression between the tomato cultivar “Heinz1706” and the wild relative *S. pimpinellifolium* indicated that *SlCCX* genes might play an important role in whole plant development in tomato. High expression levels of SlCCX1-LIKE during the flowering stages and fruit development but lower expression levels in root and leaves suggest that SlCCX1-LIKE might play a different role in the development of source-sink organs in tomato. Additionally, SlCCX5 was highly expressed in fruits, indicating that it may be involved in fruit maturation and development. Li et al. (2016) found that *AtCCX1* is expressed throughout the plant and the *Arabidopsis ccx1ccx4* doublemutant shows continuous green coloration during leaf senescence. In contrast, *AtCCX3* was expressed mainly in flowers, while *AtCCX4* was expressed throughout the *Arabidopsis* plant.

The control and maintenance of ion homeostasis is critical to all organisms (Sze et al., 2000). Cationic transport is a key process in metabolism and function, and it plays a crucial role in mineral nutrition, ionic stress tolerance, and signal transduction (Harper, 2001). AtCCX members from each group differ in substrate specificity. Functional characterization based on heterologous expression in yeast suggests that AtCCX3 and AtCCX4 from group 2 have affinity for Mn^2+^, K^+^, and Na^+^ but not for Ca^2+^ (Morris et al., 2008), while AtCCX5 from group 1 appears to mediate K^+^ uptake and Na^+^ transport but not Ca^2+^ transport (Zhang et al., 2011).

OsCCX2, from group 1, enhanced yeast tolerance to excess Na^+^, Li^+^, Fe^2+^, Zn^2+^, and Co^2+^, whereas AtCCX1, from group 3, was more resistant to low pH and high concentrations of Na^+^. In this study, the SlCCXs were spreading over different groups. The expression levels of the five SlCCXs were affected by different cationic treatments. SlCCX1-LIKE levels were much higher when exposed to MgCl_2_ or MnCl_2_. Furthermore, 8 h of exposure to NaCl and CaCl_2_ slightly increased the expression levels of SlCCX1-LIKE. The expression of SlCCX4 was dramatically increased after 24 h of exposure to CaCl_2_. In contrast, the expression of SlCCX4 was increased after only 8 h of exposure to Na^+^ and Mg^2+^. Collectively, expression and motif-related results demonstrate that SlCCXs participate in diverse cation homeostasis processes. OE-SlCCX1-LIKE plants showed normal growth before flowering, but leaf senescence was visible at around flowering, when the source/sink relationship changes dramatically, suggesting that the change in source/sink relationship may elevate the requirement for SlCCX1-LIKE activity to maintain proper cation balance.

Plant hormones have been extensively reported to regulate leaf senescence, with ethylene, abscisic acid, functioning as inducers and auxin as inhibitors (Gan and Amasino, 1997). Transcriptome data of SlCCX1-LIKE-OE plants compared with WT showed that most genes related to IAA, ABA, and ETH hormone signaling pathways were differentially expressed. Many SAUR genes are significantly downregulated in SlCCX1-LIKE-OE plants (Supplementary Table 3). In addition, our results showed two PP2C were downregulated. PP2Cs from inhibiting SnRK2s, which regulate the expression of phosphorylated transcription factors [ABA-responsive element-binding factors (ABFs)] to induce ABA-responsive SAGs, creating the yellowing symptoms of leaf senescence (Furihata et al., 2006). Ethylene has long been considered a key endogenous regulator of leaf senescence. Our results reveal that the expression of ethylene receptor, EIN3, ERF were upregulated in OE plants. Hence, SlCCX1-LIKE might induce the ABA and ETH signal to regulate their leaf mediation leaf senescence in tomato.

### SlCCX1-LIKE Regulates Leaf Senescence by Mediating Mg^2+^ Signaling and ROS Production

Normally, up to 20% of the total Mg in plants is bound in chlorophyll (Karley and White, 2009; Cakmak and Yazici, 2010). However, this proportion is able to exceed 50% in both Mg deficiency and low light conditions (Dorenstouter et al., 1985), suggesting that prior to other biological processes, Mg^2+^ is preferentially used for photosynthesis in chloroplasts. The step for chlorophyll synthesis is the insertion of Mg^2+^ into protoporphyrin IX, which is catalyzed by Mg chelatase in an ATP-dependent reaction (Willows, 2003). Mg chelatase is a heterotrimeric enzyme composed of ChII, ChID, and ChIH in plants and which utilizes Mg^2+^, ATP, and protoporphyrin IX as cofactors and substrates. Our study showed that the expression of *ChII*, *ChID*, and *ChIH* were all downregulated in OE plants, including *Solyc10g008740* (ChII) (6.35-fold), *Solyc04g015490* (ChID) (3.84-fold), and *Solyc04g015750* (ChIH) (4.53-fold) (Gibson et al., 1995; Supplementary Table 4). These results suggest that the activity of Mg chelatase has been inhibited in SlCCX-LIKE-overexpressing plants. Decreased plant chlorophyll content leads to accelerated chlorosis of transgenic plants.

According to the biological phylogenetic tree analysis, SlCCX1-LIKE and AtCCX1 belong to the same branch. *AtCCX1* could be induced by long-term Mg^2+^ deletion, and decrease of external Ca^2+^ attenuated the plant growth retardation under Mg-deficient conditions (Lenz et al., 2013). In this study, the expression of *SlCCX1-LIKE* is induced by MgCl_2_ and MnCl_2_. Genes related to of Mg^2+^ signaling (*Solyc10g008740.3* and *Solyc10g077040.2*) were decreased in OE plants. In plants, it had been reported that low manganese could eliminate the active oxygen and protect the membrane lipid from the hypoxia-induced damages (Li et al., 2012). Analysis of ROS content and related genes revealed that both were induced in SlCCX1-LIKE-OE plants. Thus, SlCCX1-LIKE maybe play a crucial role in transporting intracellular manganese to control the ROS level, affecting the leaf senescence process. In addition, ferredoxin (Fd) was recruited into the photosynthetic electron transport chain of thylakoids to mediate electron transfer from photosystem I (PSI) to a number of metabolic pathways, including NADP^+^ reduction and carbon and nitrogen assimilation. Our results showed that the expression of *Fd* was all downregulated in OE plants, including *Solyc11g006910* (1.88-fold), *Solyc10g075160* (13.4-fold), and *Solyc03g005190* (7.08-fold) (Supplementary Table 4). Nitrate is reduced to nitrite by the enzyme nitrate reductase (NR) and further reduced to ammonium ions *via* enzyme nitrite reductase (NiR) using six moles of the reduced form of ferredoxin. The activity of transgenic NR to reduce nitrite to ammonium by obtaining electrons from Fd decreased (Guan et al., 2018). Therefore, Fd and NiR may be the key factors in regulating the process of nitrogen assimilation (Davenport et al., 2015). Our results showed that the expression of ferredoxin-nitrite reductase and ferredoxin-NADP^+^ reductase were all downregulated in OE plants, including *Solyc01g108630* (12.32-fold), *Solyc10g050890* (3.3-fold), and *Solyc02g083810* (7.0-fold) (Supplementary Table 4). These results suggest that the activity of nitrogen assimilation has been inhibited in SlCCX-LIKE-overexpressing plants.

The data shown here indicate that the accelerating leaf-yellowing process observed in SlCCX1-LIKE-OE plants may be caused by the Mg^2+^ signaling and ROS homeostasis. This emphasizes the Mg deficiency affecting plant chlorophyll synthesis, which leads to generate ROS by decreasing the concentration of chlorophyll and the subsequent photosynthetic electron transfer rate and nitrogen assimilation. Hence, the higher ROS level in OE plants may modulate a variety of senescence processes through interacting with diverse pathways including hormonal regulation. Therefore, SlCCX1-LIKE regulates leaf senescence probably *via* Mg^2+^ signaling as well as modulation of ROS homeostasis.

### SlCCX1-LIKE Is Localized in the Endomembrane System

Functional epitope tags of SlCCX1-LIKE demonstrated that SlCCX1-LIKE localized to the endomembrane in plants. Furthermore, SlCCX1-LIKE appeared to localize to the plant vacuolar membrane, endoplasmic reticulum, and plasma membrane to function as a cation transporter (Figure 10). Whether SlCCX1-LIKE is localized exclusively to the *trans-*Golgi network and prevacuolar compartment remains unclear. SlCCX1-LIKE might have a role in a specific subset of excess cation uptake in OE plants because of high SlCCX1-LIKE expression levels. Furthermore, the localization of SlCCX1-LIKE to the plant vacuolar, endoplasmic reticulum, and plasma membrane, combined with its overexpression in leaves may transport different cations to regulate leaf senescence in tomato. However, the precise subcellular localization of CCX1-LIKE remains to be elucidated.

## Data Availability Statement

The datasets presented in this study can be found in online repositories. The names of the repository/repositories and accession number(s) can be found in the article/Supplementary Material.

## Author Contributions

JJ designed the experiments and edited the manuscript. JL, CC, and YZ performed the experiments. JL analyzed the data. JL and XL wrote the manuscript. All authors read and approved the final manuscript.

## Conflict of Interest

The authors declare that the research was conducted in the absence of any commercial or financial relationships that could be construed as a potential conflict of interest.

## Footnotes

1 https://solgenomics.net/plantgdb.org/

2 https://solgenomics.net/tools/blast/

3 http://www.ncbi.nlm.nih.gov/

4 https://solgenomics.net/

5 http://web.expasy.org/protparam

6 http://www.cbs.dtu.dk/services/TMHMM

7 http://smart. embl-heidelberg.de

8 https://psort.hgc.jp/form.html

9 http://gsds.cbi.pku.edu.cn/

10 http://meme.sdsc.edu/meme/meme.html

11 http://pfam.janelia.org/

12 http://smart.embl-heidelberg.de/

13 http://www.ncbi.nlm.nih.gov/Structure/cdd/wrpsb.cgi/

14 http://ted.bti.cornell.edu/cgi-bin/TFGD/digital/home.cgi

## Abbreviations

NCX: Na^+^/Ca^2+^ exchanger family
NCKX: Na^+^/Ca^2+^ K^+^ exchanger family
CCX: cation/Ca^2+^ exchanger
CAX: H^+^/cation exchanger family
CaCA: Ca^2+^/cation antiporters
IAA: auxin
ETH: ethylene
ROS: reactive oxygen species
ABA: abscisic acid
GH3: Gretchen hagen 3
SOD: superoxide dismutase
POD: peroxisome dismutase
APOD: ascorbate peroxidase
ER: endoplasmic reticulum
CDS: coding sequence
NCBI: National Center for Biotechnology Information
GRAVY: grand average of hydropathicity
TM: transmembrane
DEG: differentially expressed gene
GFP: green fluorescent protein
MEME: multiple EM for motif elicitation
NJ: neighbor-joining
OE: overexpression
MT: micro-TOM.
