# Supplementary figures and images for: Identification and Expression Profiling Analysis of the Cation/Ca2+ Exchanger (CCX) Gene Family: Overexpression of SlCCX1-LIKE Regulates the Leaf Senescence in Tomato Flowering Phase
Source: Front Genet. 2021 Jun 25;12:683904. doi: 10.3389/fgene.2021.683904 (PMC8270643; doi:10.3389/fgene.2021.683904)

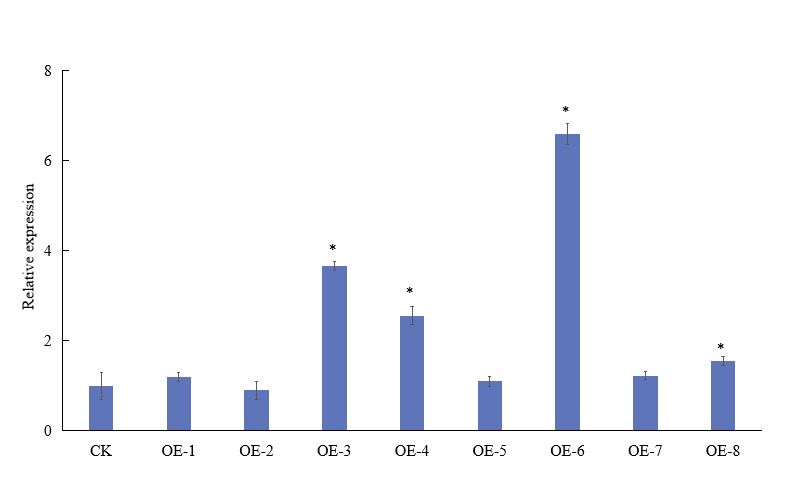

Supplement: Supplementary file 1 [file Image_1.JPEG]
