# Supplementary material for: Identification and Expression Profiling Analysis of the Cation/Ca2+ Exchanger (CCX) Gene Family: Overexpression of SlCCX1-LIKE Regulates the Leaf Senescence in Tomato Flowering Phase
Source: Front Genet. 2021 Jun 25;12:683904. doi: 10.3389/fgene.2021.683904 (PMC8270643; doi:10.3389/fgene.2021.683904)
Supplement: Supplementary file 2 [file Data_Sheet_1.doc]

**Supplement Table 1** Species and GeneBank ID in phylogenetic analysis

| Species | Gene | GeneBank ID |
| --- | --- | --- |
| *Solanum pennellii* | SpCCX1-LIKE | [XP_015063589.1](https://www.ncbi.nlm.nih.gov/protein/XP_015063589?report=genbank&log$=protalign&blast_rank=2&RID=4VP684T6014) |
|  | SpCCX4-LIKE | [XP_015080496.1](https://www.ncbi.nlm.nih.gov/protein/XP_015080496?report=genbank&log$=protalign&blast_rank=12&RID=4XS02EZF014) |
|  | SpCCX5 | [XP_015077483.1](https://www.ncbi.nlm.nih.gov/protein/XP_015077483.1?report=genbank&log$=prottop&blast_rank=1&RID=33DN6YZC014) |
| *Solanum tuberosum* | StCCX1 | [XP_015160808.1](https://www.ncbi.nlm.nih.gov/protein/XP_015160808?report=genbank&log$=protalign&blast_rank=6&RID=4VP684T6014) |
|  | StCCX1-LIKE | [XP_006343716.1](https://www.ncbi.nlm.nih.gov/protein/XP_006343716?report=genbank&log$=protalign&blast_rank=3&RID=4XS02EZF014) |
|  | StCCX4-LIKE | [XP_006342390.1](https://www.ncbi.nlm.nih.gov/protein/XP_006342390?report=genbank&log$=protalign&blast_rank=13&RID=4XS02EZF014) |
|  | StCCX5-1 | [XP_006361862.1](https://www.ncbi.nlm.nih.gov/protein/XP_006361862?report=genbank&log$=protalign&blast_rank=3&RID=4TDT5UTR014) |
|  | StCCX5-2 | [XP_015171004.1](https://www.ncbi.nlm.nih.gov/protein/XP_015171004?report=genbank&log$=protalign&blast_rank=4&RID=4TDT5UTR014) |
| *Capsicum annuum* | CaCCX1 | [PHT86360.1](https://www.ncbi.nlm.nih.gov/protein/PHT86360?report=genbank&log$=protalign&blast_rank=3&RID=4VP684T6014) |
|  | CaCCX1-LIKE | [XP_016554721.1](https://www.ncbi.nlm.nih.gov/protein/XP_016554721?report=genbank&log$=protalign&blast_rank=6&RID=4XS02EZF014) |
|  | CaCCX3-LIKE | [XP_016580161.1](https://www.ncbi.nlm.nih.gov/protein/XP_016580161?report=genbank&log$=protalign&blast_rank=18&RID=4XS02EZF014) |
|  | CaCCX5 | [PHT62826.1](https://www.ncbi.nlm.nih.gov/protein/PHT62826?report=genbank&log$=protalign&blast_rank=7&RID=4TDT5UTR014) |
| *Nicotiana sylvestris* | NsCCX1-LIKE | [XP_009758687.1](https://www.ncbi.nlm.nih.gov/protein/XP_009758687?report=genbank&log$=protalign&blast_rank=5&RID=4XS02EZF014) |
| *Dorcoceras hygrometricum* | DhCCX5 | [KZV33907.1](https://www.ncbi.nlm.nih.gov/protein/KZV33907?report=genbank&log$=protalign&blast_rank=15&RID=4TDT5UTR014) |
| *Nicotiana tabacum* | NtCCX1-LIKE | XP_016505161.1 |
|  | NtCCX3-LIKE | [XP_016433391.1](https://www.ncbi.nlm.nih.gov/protein/XP_016433391?report=genbank&log$=protalign&blast_rank=19&RID=4XS02EZF014) |
|  | NtCCX5-LIKE | [XP_016496449.1](https://www.ncbi.nlm.nih.gov/protein/XP_016496449?report=genbank&log$=protalign&blast_rank=11&RID=4TDT5UTR014) |
| *Nicotiana attenuata* | NaCCX1-LIKE | [OIT06686.1](https://www.ncbi.nlm.nih.gov/protein/OIT06686?report=genbank&log$=protalign&blast_rank=9&RID=4VP684T6014) |
|  | NaCCX4-LIKE | [XP_019245856.1](https://www.ncbi.nlm.nih.gov/protein/XP_019245856?report=genbank&log$=protalign&blast_rank=21&RID=4XS02EZF014) |
|  | NaCCX5 | [XP_019228161.1](https://www.ncbi.nlm.nih.gov/protein/XP_019228161?report=genbank&log$=protalign&blast_rank=10&RID=4TDT5UTR014) |
| *Capsicum baccatum* | CbCCX1 | [PHT52446.1](https://www.ncbi.nlm.nih.gov/protein/PHT52446?report=genbank&log$=protalign&blast_rank=5&RID=4VP684T6014) |
|  | CbCCX3 | PHT42737.1 |
|  | CbCCX5 | [PHT41142.1](https://www.ncbi.nlm.nih.gov/protein/PHT41142?report=genbank&log$=protalign&blast_rank=5&RID=4TDT5UTR014) |
| *Capsicum chinense* | CcCCX1 | [PHU11890.1](https://www.ncbi.nlm.nih.gov/protein/PHU11890?report=genbank&log$=protalign&blast_rank=10&RID=4VP684T6014) |
|  | CcCCX5 | [PHU10085.1](https://www.ncbi.nlm.nih.gov/protein/PHU10085?report=genbank&log$=protalign&blast_rank=6&RID=4TDT5UTR014) |
| *Arabidopsis thaliana* | AtCCX1 | [AT5G17860.1](http://www.arabidopsis.org/servlets/TairObject?type=gene&id=433116) |
|  | AtCCX2 | [AT3G11490](http://www.arabidopsis.org/servlets/TairObject?type=gene&id=1000690161) |
|  | AtCCX3 | [AT1G54115.1](http://www.arabidopsis.org/servlets/TairObject?type=gene&id=433961) |
|  | AtCCX4 | AT1G54115.2 |
|  | AtCCX5 | [AT1G08960.1](http://www.arabidopsis.org/servlets/TairObject?type=gene&id=32194) |
| *Solanum lycopersicum* | SlCCX1 | XP_004247508.1 |
|  | SlCCX1-LIKE | XP_010323232.1 |
|  | SlCCX4 | [CP023758.1](https://www.ncbi.nlm.nih.gov/nucleotide/CP023758.1?report=genbank&log$=nucltop&blast_rank=1&RID=33CNUF98016) |
|  | SlCCX4-LIKE | XM_004243654.3 |
|  | SlCCX5 | XP_004230178.1 |
| *Oryza sativa* | OsCCX1 | [XP_015613300.1](https://www.ncbi.nlm.nih.gov/protein/XP_015613300.1?report=genbank&log$=prottop&blast_rank=1&RID=33FD85YV014) |
|  | OsCCX2 | ABF97973.1 |
|  | OsCCX4 | [XP_015632178.1](https://www.ncbi.nlm.nih.gov/protein/XP_015632178.1?report=genbank&log$=prottop&blast_rank=1&RID=33FT38TZ016) |

**Supplement Table 2** Primers used for qRT-PCR

| Primer number | Sequence (5’-3’) | Length (bp) |
| --- | --- | --- |
| SlCCX1-F | TGTTGGGAGAGTTAGTGTGTG | 21 |
| SlCCX1-R | ACAGGGATGGTTAGCCTTC | 19 |
| SlCCX1-LIKE-F | GCCTAACTGTCCTTGCTTG | 19 |
| SlCCX1-LIKE-R | AGCCCAACTCCAAAGAACT | 19 |
| SlCCX4-F | TGGGAAATACTGCTGCTGA | 19 |
| SlCCX4-R | CCTTCTTGGGAGTGTCAATG | 20 |
| SlCCX4-LIKE-F | TGGTGCTGTAGTTGGTGGT | 19 |
| SlCCX4-LIKE-R | CATTGCCCTGCTTGATAGA | 19 |
| SlCCX5-F | CTGTCCTCTTCTTCTTCATCG | 21 |
| SlCCX5-R | TTGGGCAGTCTTGATAAGG | 19 |

Supplement Table 3 Differentilly expressed genes in hormone processes and ion transport

| Function | Ensemble ID | Description | Fold change |
| --- | --- | --- | --- |
| IAA signal/response | Solyc11g011720.1 | Auxin-induced SAUR-like protein | -6.23 |
|  | Solyc04g081270.1 | Auxin-induced SAUR-like protein | -4.20 |
|  | Solyc02g062230.1 | Auxin-induced SAUR-like protein | -5.61 |
|  | Solyc01g110580.2 | Auxin-induced SAUR-like protein | -8.45 |
|  | Solyc01g110610.3 | Auxin-induced SAUR-like protein | -5.24 |
|  | Solyc11g011650.1 | Auxin-induced SAUR-like protein | -4.85 |
|  | Solyc11g011650.1 | Auxin-induced SAUR-like protein | -2.45 |
|  | Solyc01g110570.3 | Auxin-induced SAUR-like protein | -7.32 |
|  | Solyc11g069095.1 | Auxin-induced SAUR-like protein | -6.16 |
|  | Solyc10g086200.1 | Auxin-induced SAUR-like protein | -4.58 |
|  | Solyc01g110605.1 | Auxin-induced SAUR-like protein | -6.40 |
|  | Solyc01g110590.3 | Auxin-induced SAUR-like protein | -6.33 |
|  | Solyc04g052970.2 | Auxin-induced SAUR-like protein | -5.59 |
|  | Solyc09g007970.2 | Auxin-induced SAUR-like protein | -3.88 |
|  | Solyc10g084020.1 | Auxin-induced SAUR-like protein | -3.35 |
|  | Solyc08g079150.1 | Auxin-induced SAUR-like protein | -4.81 |
|  | Solyc03g033590.1 | Auxin-induced SAUR-like protein | -6.44 |
|  | Solyc01g110710.3 | Auxin-induced SAUR-like protein | -2.85 |
|  | Solyc11g011700.1 | Auxin-induced SAUR-like protein | -4.84 |
|  | Solyc10g054720.1 | Auxin-induced SAUR-like protein | -5.12 |
|  | Solyc01g110720.2 | Auxin-induced SAUR-like protein | -2.08 |
|  | Solyc01g110630.3 | Auxin-induced SAUR-like protein | -3.03 |
|  | Solyc06g053260.1 | Auxin-induced SAUR-like protein | -4.62 |
|  | Solyc07g042470.3 | Auxin-induced SAUR-like protein | -3.78 |
|  | Solyc03g120380.3 | IAA 19 gene family | -7.61 |
|  | Solyc04g054280.2 | IAA 23 gene family | -4.28 |
|  | Solyc06g008580.3 | IAA 22 gene family | -7.04 |
|  | Solyc06g008590.3 | IAA 17 gene family | -8.17 |
|  | Solyc12g096980.2 | IAA 11 gene family | -3.40 |
|  | Solyc06g053830.3 | IAA 7 gene family | -7.11 |
|  | Solyc01g097290.3 | IAA 16 gene family | -8.18 |
|  | Solyc06g066020.3 | IAA 36 gene family | -4.12 |
|  | Solyc09g083280.3 | IAA 1 gene family | -4.02 |
|  | Solyc09g083290.3 | IAA 14 gene family | -7.67 |
|  | Solyc07g042470.3 | SlSAUR64 auxin response gene family | -3.78 |
|  | Solyc12g005310.2 | Auxin-responsive GH3-like | -8.85 |
|  | Solyc02g064830.2 | Indole-3-acetic acid-amido synthetase GH3.8 | -2.45 |
|  | Solyc02g092820.3 | Indole-3-acetic acid-amido synthetase GH3.8 | -3.53 |
|  | Solyc07g053030.3 | Indole-3-acetic acid-amido synthetase GH3.8 | -2.26 |
|  | Solyc07g054580.3 | GH3 family protein | -5.45 |
|  | Solyc07g063850.3 | Indole-3-acetic acid-amido synthetase GH3.8 | -2.45 |
| ETH signal/response | Solyc08g007230.2 | ethylene-responsive transcription factor 2 | 2.21 |
|  | Solyc08g078180.1 | ethylene-responsive transcription factor 2 | 3.48 |
|  | Solyc04g014530.1 | ethylene-responsive transcription factor 1 | 2.1 |
|  | Solyc09g066360.1 | ethylene-responsive transcription factor 1 | 3.5 |
|  | Solyc11g011750.1 | ethylene-responsive transcription factor 1 | 2.2 |
|  | Solyc01g096810.3 | ethylene-insensitive protein 3 | 2.4 |
|  | Solyc03g096630.1 | ethylene-insensitive protein 3 | 3.5 |
|  | Solyc06g053710.3 | ethylene receptor | 2.2 |
|  | Solyc09g075440.3 | ethylene receptor | 3.13 |
|  | Solyc09g089610.3 | ethylene receptor | 2.22 |
| ROS signal/response | Solyc03g122140.3 | L-lactate dehydrogenase | -3.55 |
|  | Solyc07g056540.3 | NONE | -5.56 |
|  | Solyc09g075790.3 | Long-chain fatty acid CoA ligase | -5.5 |
|  | Solyc01g079240.3 | Long-chain-fatty-acid--CoA ligase family protein | -3.5 |
|  | Solyc01g109180.3 | Long-chain-fatty-acid-CoA ligase | -2.75 |
|  | Solyc02g021140.3 | Superoxide dismutase | -2.2 |
|  | Solyc02g082760.3 | catalase | -2.6 |
| ABA signal/response | Solyc03g007230.3 | protein phosphatase 2C | 3.31 |
|  | Solyc07g040990.3 | protein phosphatase 2C | 2.67 |
| Mg^2+^-related chlorophyll synthesis | Solyc10g077040.2 | magnesium-protoporphyrin IX monomethyl ester (oxidative) cyclase | -2.6 |
|  | Solyc10g008740.3 | magnesium dechelatase | -2.2 |

Supplement Table 4 Related metabolic pathways may cause leaf yellowish

| Category | Ensemble ID | Description | Fold Change |
| --- | --- | --- | --- |
| chlorophyll-protein complex | Solyc10g008740.3 | chlI, bchI | -6.35 |
|  | Solyc04g015490.3 | chlD, bchD | -3.84 |
|  | Solyc04g015750.3 | chlH, bchH | -4.53 |
| Nitrogen metabolism pathways | Solyc01g108630.3 | ferredoxin-nitrite reductase | -12.32 |
|  | Solyc10g050890.2 | ferredoxin-nitrite reductase | -3.30 |
|  | Solyc02g083810.3 | ferredoxin--NADP^+^ reductase | -7.00 |
| chloroplast redox metabolism | Solyc11g006910.2 | ferredoxin | -1.88 |
|  | Solyc10g075160.1 | ferredoxin | -13.40 |
|  | Solyc03g005190.3 | ferredoxin | -7.08 |
| Photosystem II complexes | Solyc09g064500.3 | photosystem II 13kDa protein | -10.40 |
|  | Solyc07g054290.1 | photosystem II Psb27 protein | -4.67 |
|  | Solyc10g006530.3 | photosystem II oxygen-evolving enhancer protein 3 | -12.82 |
|  | Solyc05g007780.3 | photosystem II oxygen-evolving enhancer protein 3 | -8.62 |
|  | Solyc06g060340.3 | photosystem II 22kDa protein | -13.11 |
|  | Solyc06g084050.3 | photosystem II PsbW protein | -8.82 |
|  | Solyc06g084045.1 | photosystem II PsbW protein | -12.40 |
|  | Solyc03g114930.3 | photosystem II oxygen-evolving enhancer protein 2 | -11.73 |
|  | Solyc10g054420.2 | photosystem II oxygen-evolving enhancer protein 2 | -11.38 |
|  | Solyc09g064580.2 | photosystem II PsbM protein | -4.92 |
|  | Solyc11g006675.1 | photosystem II PsbH protein | -2.55 |
